# Association of maternal CNVs in GSTT1/GSTT2 with smoking, preterm delivery, and low birth weight

**DOI:** 10.3389/fgene.2013.00196

**Published:** 2013-10-28

**Authors:** Xiaojing Zheng, Eleanor Feingold, Kelli K. Ryckman, John R. Shaffer, Heather A. Boyd, Bjarke Feenstra, Mads Melbye, Mary L. Marazita, Jeffrey C. Murray, Karen T. Cuenco

**Affiliations:** ^1^Department of Pediatrics, School of Medicine, University of PittsburghPittsburgh, PA, USA; ^2^Department of Biostatistics, Graduate School of Public Health, University of PittsburghPittsburgh, PA, USA; ^3^Department of Human Genetics, Graduate School of Public Health, University of PittsburghPittsburgh, PA, USA; ^4^Department of Pediatrics, College of Medicine, University of IowaIowa City, IA, USA; ^5^Department of Epidemiology Research, Statens Serum InstitutCopenhagen, Denmark; ^6^Department of Oral Biology, Center for Craniofacial and Dental Genetics, School of Dental Medicine, University of PittsburghPittsburgh, PA, USA

**Keywords:** prematurity, preterm birth, copy number variation, *GSTT1*, *GSTT2*, birth weight

## Abstract

Preterm delivery (PTD) is an adverse birth outcome associated with increased infant mortality and negative lifelong health consequences. PTD may be the result of interactions between genetics and maternal/fetal environmental factors including smoking exposure (SMK). A common deletion in the *GSTT1* gene was previously reported to affect birth outcomes in smokers. In this study, we dissect the associations among SMK, birth outcomes, and copy number variations (CNVs) in the *GSTT1*/*GSTT2* region. A preterm birth case-control dataset of 1937 mothers was part of the GENEVA preterm birth study, which included genome-wide genotyping used to identify CNVs. We examined the association of SMK with birth outcomes, detected CNVs within the *GSTT1/GSTT2* region using PennCNV, and examined associations of the identified CNVs with preterm birth and with birth weight (BW) in full term birth controls, including interactions with SMK. Finally, we tested the association of CNVs in *GSTT1/GSTT2* with SMK. We confirmed the association of smoking with low BW and PTD. We identified 2 CNVs in *GSTT2* (*GSTT2*^*a*^ and *GSTT2*^*b*^), 1 CNV in *GSTTP1* and 2 CNVs in GSTT1 (*GSTT1*^*a*^ and *GSTT1*^*b*^). The *GSTT2*^*a*^ deletion was associated with reduced BW (−284 g, *p* = 2.50E-7) in smokers, and was more common in smokers [odds ratio(OR) = 1.30, *p* = 0.04]. We found that the size of the reported common deletion CNV in *GSTT1* was larger than previously shown. The *GSTTP1* and *GSTT1*^*b*^ null genotypes were in high linkage disequilibrium (LD) (*D*′ = 0.89) and less common in smokers (OR = 0.68, *p* = 0.019 and OR = 0.73, *p* = 0.055, respectively). These two deletions were in partial LD with *GSTT2*^*a*^ and *GSTT2*^*b*^ duplications. All 5 CNVs seem to be associated with increased risk of preterm birth before 35 completed weeks. CNVs in the *GSTTT1*/*GSTT2* region appear associated with low BW and PTD outcomes, but LD complicated these CNVs in *GSTT1*/*GSTT2*. In genetic association studies of BW, multiple CNVs in this region need to be investigated instead of a single polymorphism.

## Introduction

Low birth weight (LBW) refers to the weight of a newborn being less than 2500 g (Kramer, 1987) and occurs in 16% of all live-births worldwide (deOnis et al., 1998). Birth weight is regulated by two major processes: duration of gestation and intrauterine growth rate. Preterm delivery (PTD) of babies born with less than 37 weeks of gestation is responsible for one-third to two-thirds of infants with LBW (Arifeen et al., 2000; Martin et al., 2007). PTD and LBW are independent risk factors for fetal and infant mortality. However, the causes of PTD and LBW are not clear. Multiple factors may contribute to the development of LBW and/or PTD, including genetic and environmental factors, and other specific maternal-fetal characteristics (e.g., demographic, obstetric, nutritional factors, and maternal morbidity during pregnancy) (Kramer, 1987). Based on twin studies, the heritabilites of low birthweight (<2500 g) and PTD (<37 gestational weeks) are estimated to be 37–42 and 36%, respectively (Clausson et al., 2000).

One of the important environmental factors in birth outcomes is maternal tobacco smoking. Smoking exposure may reduce mean birth weight, and increase the risk of PTD and intrauterine growth restriction (Asmussen and Kjeldsen, 1975; Asmussen, 1977;, Nilsen et al., 1984; Ronco et al., 2005; Goldenberg and Culhane, 2007; Haram et al., 2007). Part of the effect of smoking on birth outcomes may be due to the metabolism of the tobacco compound PAH (polycyclic aromatic hydrocarbons) (Perera et al., 2005; Tsui et al., 2008; Wu et al., 2010). PAH is a carcinogenic compound that is detoxified in a two-stage process. PAHs are converted into procarcinogen, which is then conjugated into excretal metabolites. The conjugation is catalyzed by the product of the gene *GSTT1* (Glutathione S-transferase theta 1), which belongs to the theta class of GSTs. The class members, *GSTT1* and *GSTT2*, are located on human chromosome 22. The genes are approximately 50 kb away from each other, with a GSTT pseudo gene (*GSTTP1*) located between them. *GSTT1* and *GSTT2* share 55% amino acid sequence identity and have detoxification roles (Coggan et al., 1998), which make the GSTT1/GSTT2 region an interesting candidate for explaining smoking-induced adverse effects.

A small deletion in *GSTT1* is the only reported common copy number variant (CNV) in the region. A null genotype (homozygous deletion) of *GSTT1* modifies the effect of maternal smoking on birth outcomes. Smokers have infants with lower mean birth weight compared to non-smokers; however, the reduction varies according to the *GSTT1* copy number. Mean birth weight decreases dramatically in smokers with the *GSTT1* null genotype compared to smokers with the *GSTT1* wild-type genotype (Wang et al., 2002; Wu et al., 2007; Grazuleviciene et al., 2009; Aagaard-Tillery et al., 2010). It is unclear whether other CNVs may exist in the *GSTT1*/*GSTT2* region.

The goal of this study was to explore whether additional CNVs are distributed in *GSTT1*/*GSTT2* and to investigate their associations with smoking and birth outcomes. First, we detected CNVs in the *GSTT1*/*GSTT2* regions. Next, we examined the association of *GSTT1*/*GSTT2* CNVs with birth outcomes, stratifying by smoking status. Finally we investigated the relationship of *GSTT1*/*GSTT2* CNVs with smoking.

## Materials and methods

### Study populations

Data were generated as part of the GENEVA (Gene Environment Association studies) preterm birth study (http://www.ncbi.nlm.nih.gov/projects/gap/cgi-bin/study.cgi?study_id=phs000103.v1.p1). This study is part of dbGAP (http://www.ncbi.nlm.nih.gov/gap) (Mailman et al., 2007). A case-control study of preterm birth identified ~1000 mother-child case pairs (spontaneous births at <37 weeks of gestation), and 1000 mother-child controls pairs (spontaneous births at 40 weeks of gestation) from the Danish National Birth Cohort (DNBC) (Olsen et al., 2001). 1937 mothers had genotype information generated from the Illumina Human660W-Quad chip. Of these, 893 had a preterm birth, 978 delivered at term, and 66 were categorized as neither case nor control (births between 37 and 39 weeks of gestation) and were excluded from the current study. Smoking data came from DNBC prenatal interviews conducted at several points during gestation. For the purposes of this study, we categorized smoking as “any smoking” or “no smoking” during pregnancy.

### Genotyping and quality control

Complete genotyping and data cleaning reports for the GENEVA preterm birth study are available in dbGAP (https://www.genevastudy.org/sites/www/content/files/datacleaning/data_cleaning_reports/Preterm_Birth_DN_Murray_DCR_9-3-2010.pdf). The genotyping quality was extremely high. For this CNV study, SNPs were not filtered by minor allele frequency or Hardy-Weinberg equilibrium testing, as those filters tend to remove precisely the SNPs in CNV regions.

### CNV calls by pennCNV and identification of CNV regions

CNV calls for mothers were generated using the PennCNV software and published guidelines (2009Aug27 version) (Wang et al., 2007). A GC wave adjustment option was applied in PennCNV. We removed all samples that had been genome-wide amplified, as CNV calling in such samples is generally poor (Zheng et al., 2012). Samples were then filtered using the criterion log R ratio standard deviation (LRRSD) >0.3 resulting in 1617 out of 1937 mothers eligible for further analysis. All analyses were restricted to autosomes. Human genome build 36 was used for this study. CNVs with copy number >2 were defined as duplications, while those with copy number <2 were considered deletions. Each CNV contained at least three consecutive markers. For CNVs with high allele frequency of a deletion, we also applied the PennCNV validation algorithm.

To identify CNV regions between *GSTT1* to *GSTT2*, we first merged CNVs detected by PennCNV if the length of overlap between any two CNVs is greater than 50% of the size of at least one of the CNVs. The start position of the resulting CNV region is defined as the minimum base pair position of the overlapping CNVs, and the end position is defined to be the maximum base pair position of the overlapping CNVs.

### Statistical analysis

We examined the association of smoking with PTD and birth weight in our dataset using logistic and linear regression, respectively. The association of CNVs with birth weight was evaluated using linear regression. Since preterm birth is known to be collinear with birth weight, analyses of birth weight were performed in the controls (full term births) only. The relationship between CNVs and PTD stratified by smoking status was examined using logistic regression. The association between CNVs and smoking adjusted for preterm birth state was assessed using logistic regression.

In regression analysis, deletion CNVs [copy number (*CN) < 2*] and duplication CNVs (CN > 2) were coded as dummy variables and compared to normal copy number (*CN* = 2), for two separate comparisons. For common deletions (allele frequency of deletion >35%) in *GSTT1* and *GSTTP1*, we compared the homozygous deletions *CN* = 0 vs. *CN* > 0 as a single dichotomous comparison. We adjusted for infant sex and maternal BMI in all regression models. All calculations were completed in R (version 2.15.1) (R Development Core Team, 2009). Associations with *p* = 0.05 were considered of interest and are reported without additional correction. The linkage disequilibrium (LD) of two CNVs was measured by *D*′.

## Results

### Association between smoking and birth outcomes

The characteristics of the DNBC/GENEVA mothers by case and control status are described in Table 1. The average maternal BMI was similar between cases and controls (*p* = 0.70). As expected, the average birth weight in infants of cases was 2463 g, which is significantly lower than in controls 3719 g (*p* < 2.20E-16). The prevalence of smoking reported during pregnancy was significantly higher in cases than in controls (31.4 and 26.2% respectively, *p* = 0.029).

**Table 1:** **The characteristics of the DNBC/GENEVA mothers (case/control) plus infant birthoutcomes**.

**Maternal characteristics**	**Cases (gestational (weeks < 37) *N* = 736**	**Controls (gestational (weeks = 40) *N* = 881**	***P*-value**
Birth weight, mean (*SD*), g	2463 (650)	3719 (459)	<2.20E-16
gestational age, mean (*SD*), wk	33.9 (2.2)	40.0 (0)	
**SMOKING DURING PREGNANCY, %**			
No	68.6	73.8	0.029
Yes	31.4	26.2	
maternal BMI, mean (SD)	23.4 (4.5)	23.5 (4.0)	0.70
**INFANT SEX, %**			
Male	53.2	53.2	0.98
Female	46.8	46.8	

*N, number of mothers; SD, standard deviation; BMI, body mass index; g, grams; wk, weeks*.

We conducted association analysis of birth weight in controls (40 weeks gestation) only. Smoking was a significant predictor of birth weight (*p* = 2.01E-10). Among the infants with mothers who smoked, the mean birth weight was 277.87 g lower than in the non-smoking group.

### Identification of CNVs

**Figure 1:**
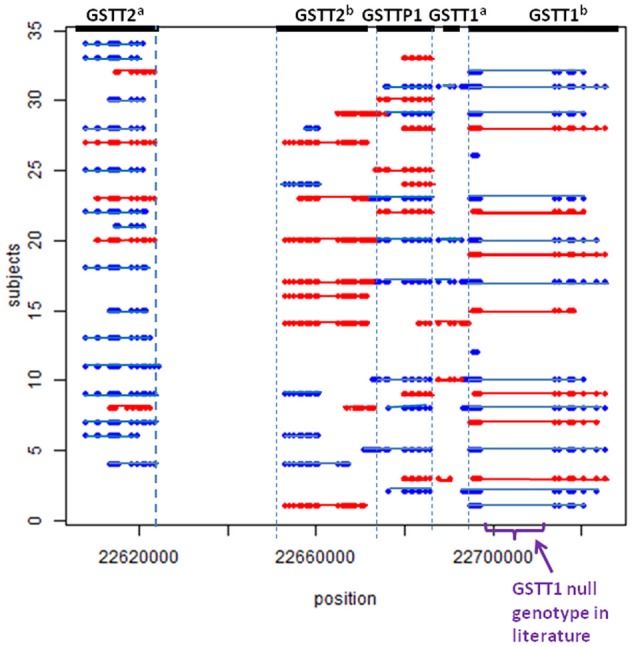
**CNV calls in a sample of 34 subjects**. Each dot represents a marker. Each line represents a CNV called by PennCNV. Red is duplication, blue is deletion. No marker was located in the region of the previously-reported *GSTT1* null genotype.

Using the methods described above, we found five potential CNV regions located in three genes in the area of *GSTT1*~*GSTT2* (Figure 1). Two CNVs were in *GSTT2* (*GSTT2*^*a*^ and *GSTT2*^*b*^), one was in *GSTTP1*, and the remaining two CNVs were in *GSTT1* (*GSTT1*^*a*^ and *GSTT1*^*b*^). These five CNVs were not independent. The duplications in *GSTT2* (*GSTT2*^*a*^ and *GSTT2*^*b*^) are correlated with two deletions, located in *GSTTP1* and *GSTT1* (*GSTT1*^*b*^). Detailed information on the relationship among these CNVs is summarized in the Table A1. None of our array markers were located within the *GSTT1* null genotype region that has previously been reported in the literature, but our *GSTT1*^*b*^ region spans that location (see Figure 1), which suggests that the deletion region is larger than previously reported.

### Association between CNVs in *GSTT1/GSTT2* and birth weight

The 5 CNVs were assessed for association with birth weight in controls (full term births) (Tables 2, 3A,B). The *GSTT2*^*b*^ deletion was the only CNV associated with birth weight in full term controls (Table 3B, 122.0 g, *p* = 0.05). The *GSTT2*^*a*^ deletion was borderline associated birthweight regardless of smoking status and when examined in solely smokers (Table 3B. in all subjects, *p* = 0.060; in smokers only, *p* = 0.07). The *GSTT2*^*b*^ deletion was also borderline associated with birthweight when looking in non-smokers only (Table 2; *p* = 0.09 among full-term births only). None of the interactions between these five CNVs with smoking were near statistical significance after evaluating a model of birthweight regressed on maternal BMI, infant sex, genotype, and genotype x smoking status (Table 2, *P*_int_).

**Table 2:** **Association of CNVs in *GSTT1*/*GSTT2* with birth weight and preterm birth**.

**Outcome**	**Full term birth only (controls)^1^**					**All subjects (cases + controls)^2^**						
	**Birth weight (g)**					**Preterm Birth**				**Very Preterm^3^ Birth**		
						**(gestational weeks <37)**				**(gestational weeks <35)**		
**GENE × SMOKING STATUS COMBINATION**												
**Genotype**	**Smk**	**N_1_**	β **(*SE*)**	***P***-**value**	**P_int_^#^**	**N_2_**	**Freq (%)**	**Odds ratio**	P^**^-value	**Freq (%)**	**Odds ratio**	***P*-value**
**GSTT2^a^**												
Normal	−	375	Ref			643	41.7	Ref		18.8	Ref	
	+	124	−162 (47)	5.90E-04		242	48.8	1.32	0.07	26.8	1.57	0.01
Duplication	−	89	20 (53)	0.70	0.87	162	45.1	1.15	0.45	24.7	1.44	0.08
	+	23	−124 (95)	0.19		50	54.0	1.55	0.13	28.0	1.68	0.12
Deletion	−	186	−20 (41)	0.62	0.18	311	40.2	0.91	0.49	19.3	1.04	0.81
	+	84	−284 (55)	2.50E-07		152	44.7	1.14	0.48	20.4	1.02	0.93
**GSTT2^b^**												
Normal	_	512	Ref			882	41.9	Ref		19.3	Ref	
	+	193	−199 (38)	2.55E-07		362	46.7	1.21	0.15	24.3	1.29	0.09
Duplication	−	91	25 (51)	0.62	0.45	163	44.2	1.09	0.61	25.8	1.48	0.05
	+	23	−90 (94)	0.33		51	54.9	1.67	0.08	31.4	1.86	0.05
Deletion	−	45	117 (69)	0.09	0.68	69	34.8	0.63	0.09	13.0	0.58	0.16
	+	14	−141 (119)	0.24		29	51.7	1.47	0.31	17.2	0.85	0.74
**GSTTP1**												
Present	−	544	Ref			928	41.4	Ref		18.8	Ref	
	+	204	−204 (37)	6.11E-08		390	47.7	1.31	0.03	24.6	1.35	0.04
Null	−	106	47 (47)	0.32	0.62	188	43.6	1.11	0.53	24.6	1.38	0.09
	+	27	−105 (88)	0.23		54	50.0	1.31	0.35	25.9	1.48	0.24
**GSTT1^a^**												
Normal	−	570	Ref			974	41.5	Ref		20.2	Ref	
	+	199	−213 (37)	1.27E-08		388	48.7	1.34	0.02	25.5	1.29	0.07
Duplication	−	26	−102 (89)	0.25	0.11	51	49.0	1.23	0.46	15.7	0.62	0.26
	+	13	−62 (129)	0.63		22	40.9	1.05	0.90	18.2	0.89	0.83
Deletion	−	54	61 (65)	0.34	0.87	91	40.7	0.97	0.88	17.6	0.87	0.62
	+	19	−131 (109)	0.23		34	44.1	1.07	0.85	20.6	0.95	0.92
**GSTT1^b^**												
Present	−	543	Ref			927	41.4	Ref		18.7	Ref	
	+	202	−200 (37)	1.12E-07		386	47.7	1.31	0.03	24.6	1.35	0.04
Null	−	107	57 (47)	0.23	0.83	189	43.4	1.09	0.58	24.9	1.42	0.06
	+	29	−121 (85)	0.15		58	50.0	1.32	0.33	25.9	1.48	0.22

*Marginal effects of CNVs and joint effects of CNV with smoking status reported*.

1 *Mothers with full term births. These subjects are known as “controls” and used for the All subjects study sample*.

2 *Cases (preterm or very preterm birth mothers) and “controls”^3^. Research definition of preterm birth*.

3 *Ref is the subgroup designated as the referent group for comparison with various CNV subgroups*.

a and b *were used to distinguish different CNVs in the same gene*.

*Smk is mother reported no smoking (−) or smoking (+) during pregnancy*.

*N_1_ is the number of mothers with full-term birth only*.

*N_2_ is the total number of mothers (cases + controls)*.

*Freq (%) is the percentage of N_2_ with infant births of either <37 or <35 gestational weeks*.

*β (SE) is the regression coefficient and standard error estimate for the model under evaluation*.

*P-value is from the model of Outcome = α + maternal BMI + infant sex + (CNV + SMK). Each combination of genotype and smoking status was compared to subjects with normal CNV genotype and who were non-smokers*.

*P_int_ is the p-value for the interaction term in the model is Outcome = α + maternal BMI + infant sex + SMK + CNV + CNV × SMK*.

**Table 3:** **Effect of CNVs in *GSTT1/GSTT2* region on birth weight in full term birth mothers^∧^**.

**(A) Duplication**									
				**All^1^**		**Smokers**		**Non-smokers**	
**Gene symbol**	**Start position**	**End position**	**No. of SNPs^2^**	**Change of mean BW^3^**	***P*-value^4^**	**Change of mean BW**	***P*-value**	**Change of mean BW**	***P*-value**
*GSTT2*^a^	22613516	22623656	11	25.1	0.60	38.09	0.71	12.2	0.82
*GSTT2*^b^	22653131	22671429	30	45.5	0.32	98.31	0.33	13.9	0.78
*GSTTP1*	22679906	22685981	10	−			−	−	−
*GSTT1*^*a*^	22693154	22694601	17	−51.5	0.50	122.58	0.35	−113.8	0.20
*GSTT1*^b^	22695592	22723364	23	−			−	−	−
**(B) Deletion**									
				**All**		**Smokers**		**Non-smokers**	
**Gene symbol**	**Start position**	**End position**	**No. of SNPs**	**Change of mean BW**	***P*-value**	**Change of mean BW**	***P*-value**	**Change of mean BW**	***P*-value**
*GSTT2*^a^	22613516	22623656	11	−65.3	0.06	−116.8	0.07	−24.9	0.54
*GSTT2*^b^	22653131	22671429	30	122.0	0.05	91.1	0.47	120.2	0.08
*GSTTP1*	22679906	22685981	10	66.7	0.12	92.1	0.33	41.1	0.39
*GSTT1*^a^	22693154	22694601	17	80.3	0.15	33.4	0.76	97.4	0.13
*GSTT1*^b^	22695592	22723364	23	68.6	0.11	79.8	0.38	49.7	0.29

*Duplication (A) and deletion (B) effects*.

∧ *“Full term birth mothers” are designated “controls” elsewhere*.

1 *“All” consists of Smoker and Non-smoker mothers combined*.

2 *“No. of SNPs” is the number of SNPs typed within the region defined by start and end positions*.

3 *“Change of mean BW” is the amount of birthweight (in grams) altered that is attributed to the CNV. This value is obtained from a regression coefficient estimate for the CNV in the model while adjusting for maternal body mass index and infant sex*.

*“P-value” is the p-value associated with change of mean BW*.

*−No estimate obtained*.

### Association between CNVs in *GSTT1/GSTT2* and preterm birth

We then analyzed the association of CNVs with preterm birth (gestational weeks <37) and with birth <35 gestational weeks (Table 2). None of the CNVs were significantly associated with the risk of preterm birth among smoking and non-smoking mothers considered separately (Table 4). However, the *GSTT2*^*a*^ duplication was borderline significantly associated with an increased risk of birth <35 weeks (odds ratio = 1.44, *p* = 0.08) in non-smokers (Table 2). Among smokers, the duplication did not significantly influence the risk of birth <35 weeks (Table 4; odds ratio = 1.06, *p* = 0.88). The *GSTT2*^*a*^ deletion was associated with reduced birth weight in full term controls with borderline significance when smoking status was not considered (Table 3B; −65.3 g, *p* = 0.06), but was not associated with preterm birth or birth before 35 weeks (Table 2). Three CNVs [*GSTT2*^*b*^ duplication (odds ratio = 1.48, *p* = 0.05), *GSTTP1* deletion (odds ratio = 1.38, *p* = 0.09) and *GSTT1*^*b*^ deletion (odds ratio = 1.42, *p* = 0.06)] were all independently associated or borderline associated with birth <35 weeks in non-smokers (Table 2). These three CNVs were also in partial LD with each other. The deletions in *GSTTP1* and *GSTT1*^*b*^ are common and the homozygous deletion (null genotypes) frequencies are 15.5 and 15.8% respectively. The duplication of *GSTT2*^*b*^ when combined with the effect of smoking was similar to the CNV's effect among non-smokers (Table 2: odds ratio = 1.86, *p* = 0.05). The deletion of *GSTT1*^*b*^ was associated with increased risk of birth before 35 weeks (odds ratio = 1.42, *p* = 0.06) in non-smokers and when combined with smoking had comparable effect but was not statistically significant as joint risk factors (Table 2. odds ratio = 1.48, *p* = 0.22). The marginal effect in smokers was not significant either (Table 4B: odds ratio = 1.09, *p* = 0.80). The odds ratio for the effect of the *GSTTP1* deletion on birth before 35 weeks was similar [Table 2: odds ratio = 1.38 (*p* = 0.09) and 1.48 (*p* = 0.24), respectively, in non-smokers and when considered jointly with smoking] to that of the *GSTT1*^*b*^ deletion.

**Table 4:** **Association of CNVs in the *GSTT1/GSTT2* region with preterm and very preterm delivery in mothers stratified by smoking status^∧^**.

**(A) Duplication**										
	**Smokers**						**Non-smokers**			
**Gene symbol**	**No. of CNVs in PTD mothers (*N* = 213)**	**No. of CNVs in Non-PTD mothers (*N* = 231)**	**PTD (gestational weeks <37)**		**Very Preterm^1^ Birth (gestational weeks <35)**		**No. of CNVs in PTD mothers (*N* = 466)**	**No. of CNVs in Non-PTD mothers (*N* = 650)**	**PTD (gestational weeks <37)**	
			**Odds ratio**	***P*-value**	**Odds ratio**	***P*-value**			**Odds ratio**	***P*-value**
*GSTT2*^a^	27	22	1.23	0.50	1.06	0.88	70	89	1.14	0.44
*GSTT2*^b^	28	23	1.37	0.29	1.42	0.28	72	91	1.12	0.49
*GSTTP1*	−	−	−	−			−	−	−	−
*GSTT1*^a^	9	13	0.73	0.48	0.68	0.51	25	26	1.36	0.29
*GSTT1*^b^	−	−	−	−				−	−	−
**(B) Deletion**										
	**Smokers**						**Non-smokers**			
**Gene Symbol**	**No. of CNVs in PTD mothers (*N* = 213)**	**No. of CNVs in Non-PTD mothers (*N* = 231)**	**PTD (gestational weeks <37)**		**Very Preterm Birth (gestational weeks <35)**		**No. of CNVs in PTD mothers (*N* = 466)**	**No. of CNVs in Non-PTD mothers (*N* = 650)**	**PTD (gestational weeks ^∧^37)**	
			**Odds ratio**	***P*-value**	**Odds Ratio**	***P*-value**			**Odds ratio**	***P*-value**
*GSTT2*^a^	68	84	0.85	0.44	0.65	0.10	125	186	0.94	0.66
*GSTT2*^b^	15	14	1.23	0.58	0.99	0.54	24	45	0.71	0.19
*GSTTP1*	26	28	1.10	0.75	1.09	0.80	75	96	1.10	0.57
*GSTT1*^a^	15	19	0.83	0.61	0.68	0.50	37	54	0.97	0.88
*GSTT1*^b^	32	34	1.10	0.74	1.09	0.80	93	120	1.08	0.62

*Duplication (A) and deletion (B) effects*.

∧ *Comparisons are made between each CNV and normal copy number as the referent group*.

*N is number of mothers*.

*No. is number*.

*PTD is preterm delivery*.

1 *Research definition of preterm birth*.

*−No estimate obtained*.

### Association between *GSTT1/GSTT2* and smoking

To better understand the relationship among birth outcomes, smoking, and the CNVs we examined, we also tested association between the CNVs and smoking. Our examination of the relationship between smoking and *GSTT1*/*GSTT2* CNVs while adjusting for birth weight indicated that the duplication CNVs were not significantly associated with smoking. However, the deletion CNV in *GSTT2*^*a*^ was significantly associated with smoking (Table 5. odds ratio of smoking vs. non-smoking in carriers of this deletion = 1.30, *p* = 0.04). This same deletion and smoking were jointly associated with decreased birth weight (Table 2; −284 g, *p* = 2.50 × 10E-07), but the deletion alone accounted for less than half of the decreased weight (Table 3B. −116.8 g, *p* = 0.07) in smokers. Smokers were also less likely to have the deletions in *GSTTP1* and *GSTT1*^*b*^ (Table 5: odds ratios = 0.68 (*p* = 0.02) and 0.73 (*p* = 0.06), respectively). These deletions tend to increase birth weight in controls, although not significantly (Table 2).

**Table 5:** **Association of CNVs in the region from *GSTT1/GSTT2* region with smoking^∧^**.

			**Duplication**		**Deletion**	
**Gene symbol**	**Start position**	**End position**	**Odds ratio**	***P*-value**	**Odds ratio**	***P*-value**
*GSTT2*^a^	22613516	22623656	0.82	0.27	1.30	0.04
*GSTT2*^b^	22653131	22671429	0.77	0.12	0.68	0.20
*GSTTP1*	22679906	22685981	−	−	0.68	0.02
*GSTT1*^a^	22693154	22694601	1.08	0.76	0.94	0.76
*GSTT1*^b^	22695592	22723364	−	−	0.73	0.06

∧ *Comparison of CNV with smoking status adjusting for birth weight*.

*−No estimate obtained*.

## Discussion

Linkage and SNP association studies have identified some genetic factors that are associated with adverse birth outcomes, including *GSTT1*. However, few CNV studies have been conducted for LBW and PTD outcomes. We focused on examining CNVs in the region from *GSTT1* to *GSTT2* and investigating their association with adverse birth outcomes and smoking.

Our finding that smoking may be associated both preterm birth and birth weight is consistent with other reports (Horta et al., 1997; Chan et al., 2001).

After identifying five CNV regions in *GSTT2, GSTTP1*, and *GSTT1*, we noted the complex association patterns with preterm birth and birth weight, which may be related to LD among the CNVs. The *GSTT2* duplications *GSTT2*^*a*^ and *GSTT2*^*b*^), *GSTTP1* deletion and *GSTT1*^*b*^ deletion are in partial LD (see Table A1). The *GSTT2*^*a*^ deletion decreased the mean birth weight in infants of smoking mothers and is more likely to be present in smokers, but the weight change was of borderline statistical significance. In contrast, the *GSTT2*^*b*^ deletion increases the birth weight (Table 3B: 120.2 g, *p* = 0.08) in infants of non-smoking mothers and is more common in non-smokers (Table 5: odds ratio of smoking vs. non-smoking = 0.68, *p* = 0.20), but again the weight change was of borderline statistical significance. Interestingly, neither of the *GSTT2* deletions was associated with preterm birth or “very preterm” birth (<35 weeks) (Windham et al., 2000; Danileviciute et al., 2012), but both *GSTT2* duplications appear to be associated with increased risk of birth before 35 weeks in non-smokers, and associated with an even higher risk when smoking and the duplications were considered jointly. These findings suggest that *GSTT2* may have a role in detoxification of tobacco, although it is also possible that these results are explained by LD with other polymorphisms or multiple testing effects. CNVs in *GSTT2* may influence the metabolism of nicotine, and exacerbate the toxic effects of smoking.

The deletions in *GSTTP1* were in high LD with *GSTT1*^*b*^ (22,695,592–22,725,333 bp) (*D*′ = 0.89) and in moderate LD with two duplications in *GSTT2* (*D*′ = 0.62). We did not observe significant effects of *GSTTP1* and *GSTT1*^*b*^ deletions on birth weight in full term controls, but they were associated with increased risk of birth before 35 weeks in both non-smokers and smokers, with the odds ratio slightly higher when the deletions were considered jointly with smokers. This suggests that *GSTTP1* and *GSTT1*^*b*^ deletions are associated with reduced birth weight through very preterm birth, but the effect on birth weight was not detected when including full term infants. We also found that *GSTTP1* and *GSTT1*^*b*^ deletions were less likely to occur in smokers. Smoking could be a proxy for some other linked yet unknown behavior or may be independently associated with these deletion CNVs.

We compared our CNV results in *GSTT1* and *GSTT2* with published findings for the previously described *GSTT1* CNV, which was reported to modify the effect of smoking on birth weight (Wang et al., 2002; Wu et al., 2007; Grazuleviciene et al., 2009; Aagaard-Tillery et al., 2010). Smokers with the null genotype of *GSTT1* had infants with lower mean birth weight than those with the wildtype genotype of *GSTT1*. The deletion's physical genomic position (22,698,742–22,706,887 bp on chromosome 22) was not covered by the HumanHap660 chip resulting in a marker gap between 22,697,104, and 22,713,954 bp in our arrays. However, our *GSTT1*^*b*^ deletion encompasses the reported *GSTT1* null genotype region. It is notable that the frequency of the homozygous *GSTT1*^*b*^ deletion CNV in our data was highly consistent with the previously reported frequency of the *GSTT1* null genotype in Denmark (15%) (Saadat et al., 2001). In contrast to most previous studies, we were able to examine the influence of *GSTT1*^*a*^ and *GSTT1*^*b*^ deletions on fetal growth in women with full term births, which means that we were able to examine the effects on birth weight more or less independently of effects on gestational age. We did not find significant association of *GSTT1* deletions with birth weight in full term birth, nor did we observe interactions of genotypes and smoking status on birth weight in controls. This may be due to the relatively small number of mothers who smoked during pregnancy, or due to the smoking measurement being a dichotomous measure. When contrasted with earlier reports of a GSTT1—smoking interaction impacting birth outcomes, the current study's lack of evidence for an interaction may be attributed to differences in confounders included in models, variability of smoker definitions, and this study's mothers being categorized as non-smokers based on the pregnancy time period only.

A single SNP (rs1622002) in *GSTT2* has been associated with metabolism of major tobacco carcinogen PAH (Wang et al., 2008), but this SNP was not included in the present study's genotype panel.

There are several epidemiologic limitations in our study. Smoking was recorded as any smoking present during pregnancy, which did not consider the influence of smoking in different trimesters. Secondly, regressions were adjusted for some demographic factors, but excluded adjustments for psychosocial factors. Statistical evaluations were not corrected for multiple testing which tempers the conclusions that can be drawn about associations, but the uncorrected *p*-values do suggest a prioritization of findings to pursue in future studies.

In addition to the epidemiological issues, CNV calls are notoriously error-prone. It is not feasible to validate specific CNV calls within the context of a large-scale association study. However, for any given CNV with calling error rates independent of phenotype, those errors should only reduce power and not cause bias in the association measure (Zheng et al., 2012). We have no reason to expect CNV calling biases in the current study, so we feel that our results are quite robust. Moreover, the fact that our frequency for the *GSTT1*^*b*^ homozygous deletion was an almost perfect match to the frequency reported in this population for the known deletion was very reassuring. We did not compare our PennCNV CNV calls to any other algorithm in this study, although we did so previously in a more explicit algorithm comparison (Zheng et al., 2012).

Despite these limitations, our study is able to shed light on CNVs in smoking-associated adverse birth outcomes. Our candidate gene analysis in *GSTT1/GSTT2* and identification of five CNVs that appear to be associated with birth weight and/or birth before 35 weeks is novel. The distribution and LD pattern of these CNVs are more complicated than expected, which may impact our understanding of the relationship between the *GSTT1* deletion and adverse birth outcomes. Follow-up genetic association studies of birth weight need to include multiple CNVs in this region instead of single polymorphisms. Additional studies including molecular evidence will be needed to validate the detected CNVs and replicate these results.

### Conflict of interest statement

The authors declare that the research was conducted in the absence of any commercial or financial relationships that could be construed as a potential conflict of interest.

## Figures and Tables

**Table A1:**
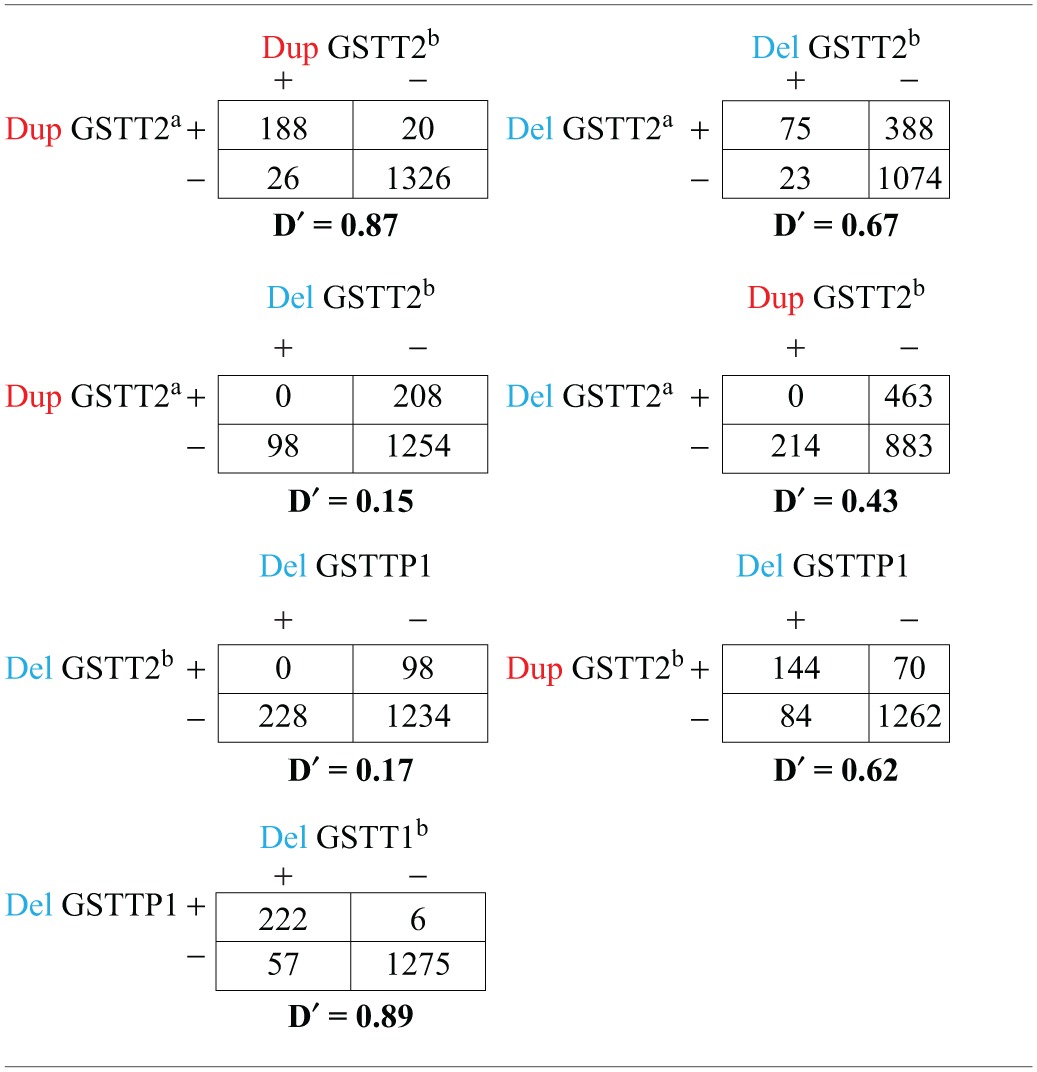
**Cross-tabulation and linkage disequilibrium of CNVs in *GSTT1/GSTT2* region. Duplication (Dup) or deletion (Del) is present (+) or absent (−)**.
